# Pulmonary Cryptococcosis in Patient with COVID-19: A Case Report

**DOI:** 10.1007/s44229-023-00030-5

**Published:** 2023-03-08

**Authors:** Sirine Ahmad, Mohammed Alsaeed

**Affiliations:** 1 Medicine Department, Dr. Sulaiman Al Habib Medical Group, Riyadh, Saudi Arabia; 2 Infectious Disease Division, Medicine Department, Prince Sultan Military Medical City, P.O.Box 7897, Riyadh, 11159 Saudi Arabia; 3 College of Medicine, Alfaisal University, Riyadh, Saudi Arabia

**Keywords:** COVID-19, SARS-CoV-2, *Cryptococcus neoformans*, Corticosteroids, Tocilizumab

## Abstract

Opportunistic infections are well known complications of COVID-19 (SARS-CoV-2 infection). Coinfections with fungi are prevalent, with aspergillus and candida infections predominating. However, the incidence of cryptococcosis related to COVID is on the rise. Here, we present a case of an 87-year-old man with COVID-19-associated pulmonary cryptococcosis. While corticosteroids were initiated upon his presentation and tocilizumab added during the disease, his clinical state deteriorated to the point where he needed mechanical ventilation. He had a positive tracheal culture for *Cryptococcus neoformans*. An antifungal medication was administered. Unfortunately, he passed away. Cryptococcal infection is uncommon in the immunocompetent population. Reported cases show an increase in the incidence of cryptococcosis in COVID-19-infected patients, which may be attributable to SARS-CoV-2 infection itself and/or immunomodulatory medications.

## Introduction

Severe acute respiratory syndrome coronavirus-2 (SARS-Cov-2) is highly associated with secondary infections in critically ill patients [1]. Recent studies have been conducted to determine the risk factors in such patients [2]. It was found that an impaired immune system and the use of immunomodulatory therapies both play important roles in bacterial and fungal coinfections in patients with COVID-19 [3, 4]. In addition, other factors, such as respiratory failure, lymphopenia, and admission to the intensive care unit, have been identified [5]. Recognizing these potential complications is crucial since secondary infections have a significant mortality risk, especially in immunosuppressed individuals. In this article, we discuss the case of a patient with COVID-19 who developed cryptococcosis pneumonia while receiving treatment with high-dose corticosteroids and tocilizumab.

## Case Presentation

**Fig. 1:**
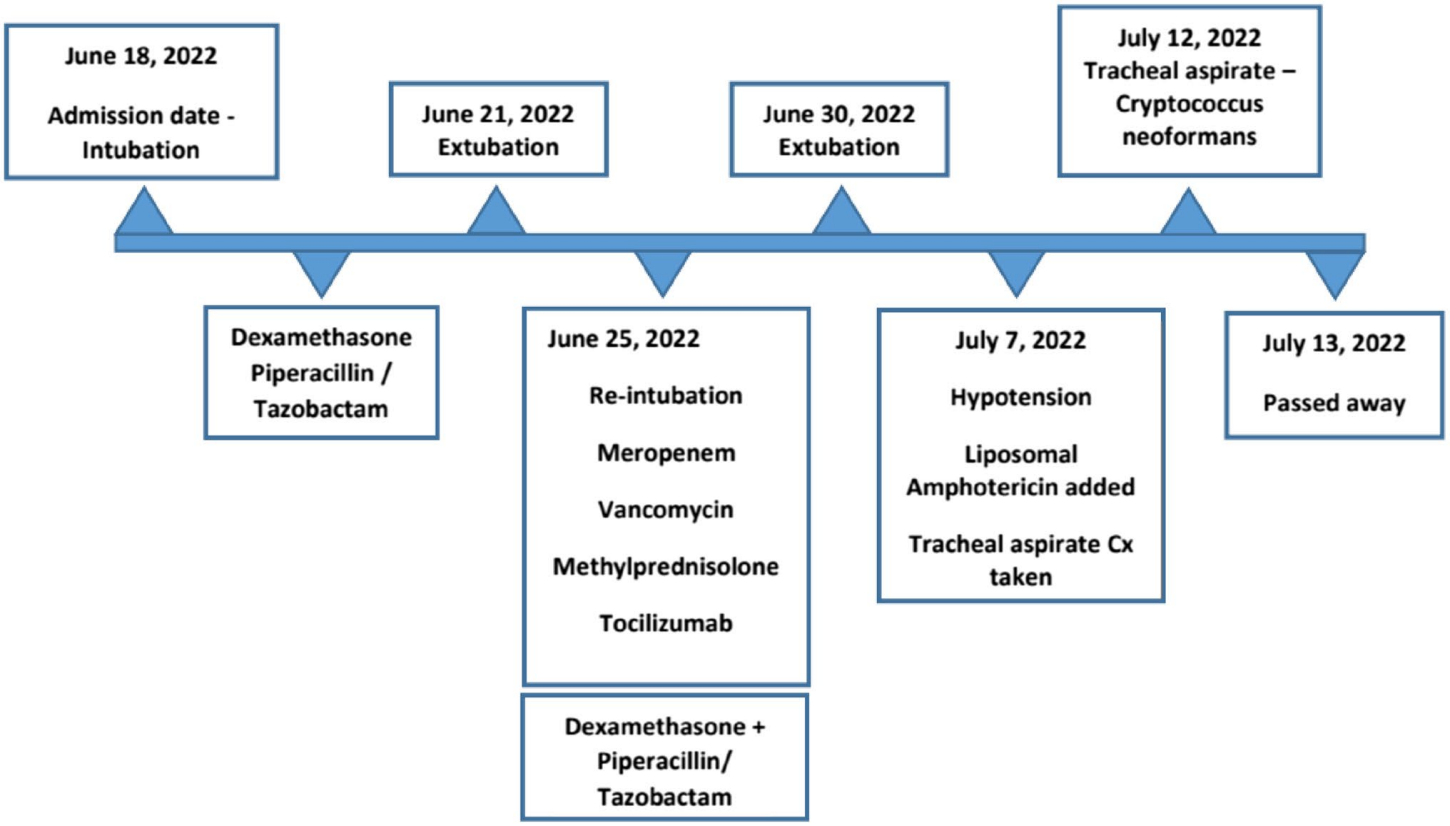
Course of disease during admission

**Fig. 2:**
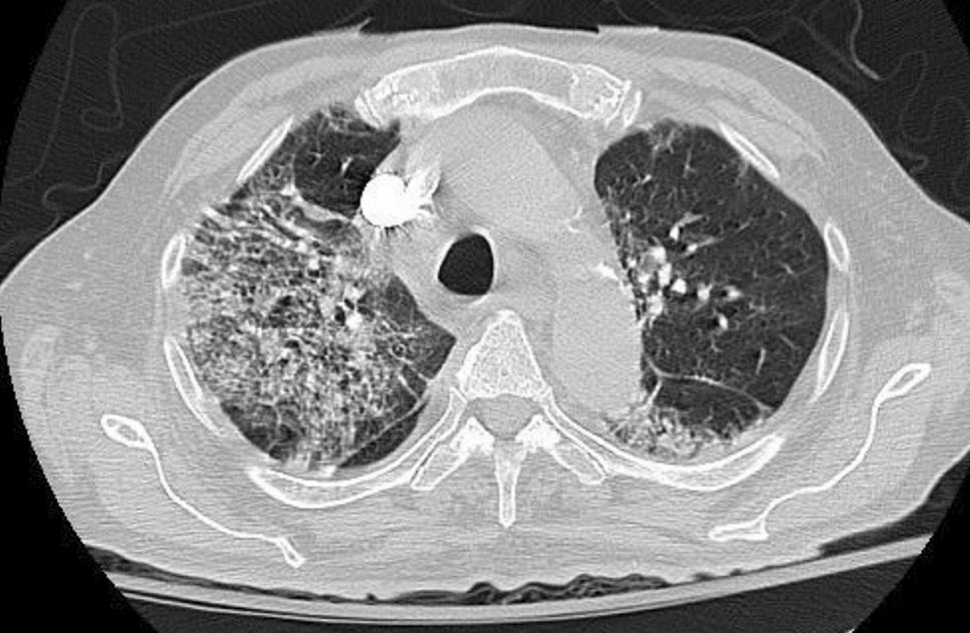
CT of chest upon admission

We present a case of an 87 year-old male with no prior known medical conditions who presented to the emergency room on June 18, 2022, complaining of shortness of breath, cough, and fever that had been present for 2 weeks (Fig. 1). At the time of the presentation, oxygen saturation was 79%, with no improvement with high-flow oxygen therapy. In addition, inflammatory markers were found to be elevated. Leukocyte count was 22.44 (reference range between 4.00 and 11.00), total neutrophil count 16 (reference range between 2.0 and 7.0), total lymphocyte count 2 (0.6–4.0), CRP 149 mg/l (reference range < 5), d-dimer 1.9 mg/l (reference range 0.0–0.5), and ferritin 2000 ng/ml (20–400). CT-chest showed patchy consolidation, bilateral interlobular septal thickening, and ground glass opacities (Fig. 2).

The test for COVID-19 PCR turned positive. Therefore, dexamethasone, 6 mg intravenously once daily, and piperacillin/tazobactam, 4.5 g IV every 6 h, was started. On June 21, the patient was extubated because of his clinical improvement.

He was hemodynamically stable until June 25, when his oxygen level dropped to 77%, and re-intubation was again required. A chest x-ray revealed bilateral infiltrates and a right-sided pneumothorax.

A chest tube was inserted. Tazobactam/piperacillin was stopped, a septic workup was sent, and meropenem and vancomycin were given. Dexamethasone was discontinued, and 80 mg of methylprednisolone was administered IV twice daily. The patient, who tested negative for HIV, HBV, and HCV, received two doses of tocilizumab (8 mg/kg each). Blood and tracheal cultures were reported as negative. He started to improve clinically and was extubated on June 30.

**Fig. 3:**
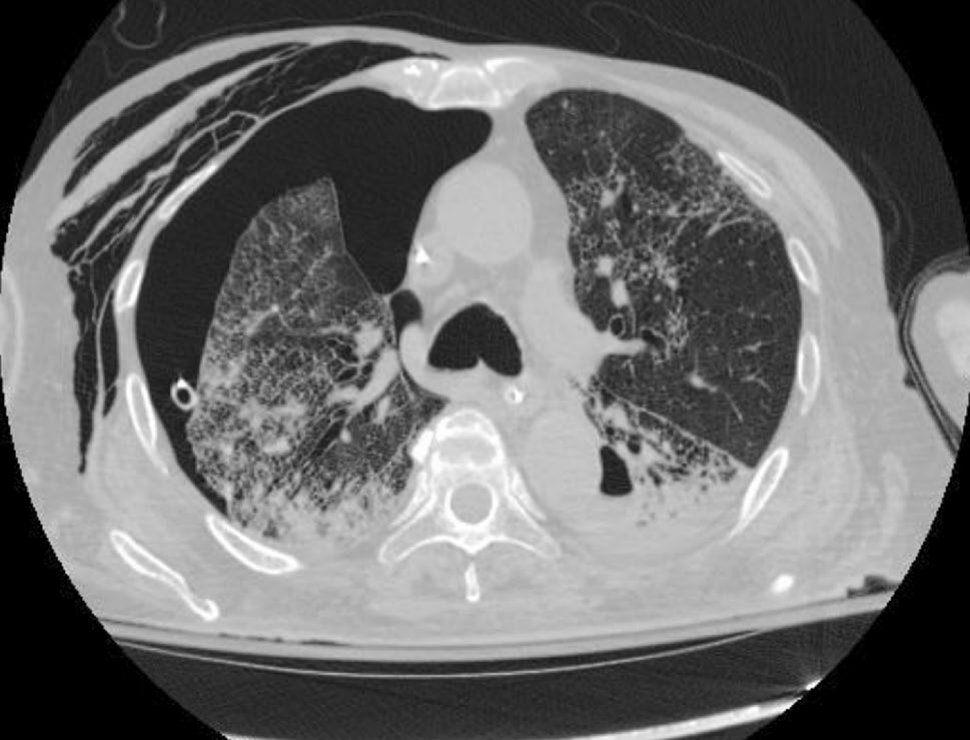
Repeated CT of chest

On July 9, while the patient was still on broad-spectrum antibiotics, he desaturated and became hypotensive with no response to IV fluid. He was reintubated and started on vasopressor. A high-resolution chest CT scan revealed bilateral reticular infiltrates with interval cavity development with the air-fluid level at the posterior paramedian aspect of the left lung (Fig. 3).

Tracheal aspirate samples were sent for fungal and mycobacterial cultures, and antifungal treatment was initiated. Fungi culture revealed the growth of *Cryptococcus neoformans* a few days later, while TB PCR and staining were negative. Unfortunately, despite receiving liposomal amphotericin, the patient died on July 13.

## Discussion and Conclusion

Critically ill patients with COVID-19 are strongly associated with secondary fungal infections, increasing morbidity and mortality [6]. Aspergillosis, candidiasis, cryptococcosis, and mucormycosis are the most common fungal diseases associated with COVID-19 (SARS-CoV-2 infection) [2]. The main causes of opportunistic infections are physiological factors such as high levels of proinflammatory cytokines, decreased T lymphocyte count, and the use of immunomodulatory medications (corticosteroids and tocilizumab) [3, 4, 6].

A reduction in COVID-19 mortality using dexamethasone was demonstrated by previous studies [7, 8]. In addition, suppression of the immune system, lymphocyte proliferation, and cytokine level reduction was linked to the described outcomes. However, a prolonged course of corticosteroid medication may result in opportunistic infections like cryptococcus [9]. In addition, the literature highlights the potential for even immunocompetent hosts to develop fungal infections when receiving corticosteroid therapy to treat COVID-19 [10].

Importantly, a review by Amin et al. discussed the risk factors for subsequent fungal infections in patient with COVID-19, including low oxygen levels, diabetes mellitus, steroid use, acidosis, high ferritin levels, mechanical ventilation, and lymphopenia [2].

*Cryptococcus neoformans* is a yeast-like fungus that causes disease in immunocompromised individuals. High-risk factors for cryptococcal infection include HIV infection with CD4 counts fewer than 200 cells/mm^3^, organ transplantation, corticosteroids use, and immune-modulating therapy. In addition, a person can become infected by inhaling contaminated soil spores, which can then spread the disease hematogenously to other organs, especially in immunocompromised people [11]. Therefore, the subsequent course of the disease is most dependent on the immunological state.

In March 2022, a literature review on cryptococcal infection in patient with COVID-19 was published. Thirteen patients were reported to have been diagnosed with a cryptococcal infection. Ten received only corticosteroids, two received both corticosteroids and tocilizumab, and one received tocilizumab alone [12]. The median interval between COVID-19 and Cryptococcus infection was thirteen days, and two individuals were diagnosed postmortem. Seven patients had a pulmonary cryptococcal infection, while four had cryptococcal meningitis. Two patients had disseminated cryptococcosis with lung and meningeal involvement. Seven patients died.

In another multicenter research network study, Chastain et al. investigated the incidence and risk variables linked to cryptococcosis in patients with COVID-19 [13]. Sixty-five cases of cryptococcosis were recorded among 212,479 hospitalized patients with COVID-19 between February 2022 and April 2022. The incidence of cryptococcal disease after COVID-19 was 0.022%, with males being more susceptible. Interestingly, corticosteroids were not shown to increase the incidence of cryptococcosis. Patients with cryptococcosis were 19 times more likely to have been given tocilizumab and 12 times more likely to have been given baricitinib than those without cryptococcosis.

Although cryptococcal infection is uncommon in the immunocompetent population, the reported cases show an increase in the incidence of cryptococcosis in patients with COVID-19, which may be attributable to SARS-CoV-2 infection itself and/or immunomodulatory medications. As a result, clinicians should suspect this opportunistic infection in a patient with COVID-19 whose clinical condition worsens after receiving immunomodulatory therapies.

## Data Availability

The datasets used during the current study are available from the corresponding author upon request.

## Abbreviations

CT: Computed tomography
HIV: Human immunodeficiency virus
COVID-19: Coronavirus disease of 2019
HBV: Hepatitis B virus
HCV: Hepatitis C virus
TB: Tuberculosis
